# Consistency and Variability in Children’s Word Learning Across Languages

**DOI:** 10.1162/opmi_a_00026

**Published:** 2019-06

**Authors:** Mika Braginsky, Daniel Yurovsky, Virginia A. Marchman, Michael C. Frank

**Affiliations:** Department of Brain and Cognitive Sciences, Massachusetts Institute of Technology; Department of Psychology, University of Chicago; Department of Psychology, Stanford University; Department of Psychology, Stanford University

**Keywords:** word learning, language acquisition, corpus analysis

## Abstract

Why do children learn some words earlier than others? The order in which words are acquired can provide clues about the mechanisms of word learning. In a large-scale corpus analysis, we use parent-report data from over 32,000 children to estimate the acquisition trajectories of around 400 words in each of 10 languages, predicting them on the basis of independently derived properties of the words’ linguistic environment (from corpora) and meaning (from adult judgments). We examine the consistency and variability of these predictors across languages, by lexical category, and over development. The patterning of predictors across languages is quite similar, suggesting similar processes in operation. In contrast, the patterning of predictors across different lexical categories is distinct, in line with theories that posit different factors at play in the acquisition of content words and function words. By leveraging data at a significantly larger scale than previous work, our analyses identify candidate generalizations about the processes underlying word learning across languages.

## INTRODUCTION

Despite tremendous individual variation in children’s rate of development (Fenson et al., 2007), the first words that they utter are strikingly consistent (Schneider, Yurovsky, & Frank, 2015; Tardif et al., 2008): they tend to talk about important people in their life (“mom,” “dad”), social routines (“hi,” “uh-oh”), animals (“dog,” “duck”), and foods (“milk,” “banana”). Even as children learn from their own experiences and according to their own interests (Mayor & Plunkett, 2014; Nelson, 1973), their vocabulary grows rapidly, typically adding more nouns, but also verbs (“go”) and other predicates (“hot”) to their repertoires. Over just their first 3 years, children learn hundreds, even thousands of words (Fenson et al., 1994; Mayor & Plunkett, 2011).

One classic approach to word learning focuses on the specific mechanisms that children bring to bear on the learning problem. For example, across many laboratory experiments, a variety of mechanisms have been identified as plausible drivers of early word learning, including co-occurrence based and cross-situational word learning (Schwartz & Terrell, 1983; Yu & Ballard, 2007), social cue use (Baldwin, 1993), and syntactic bootstrapping (Gleitman, 1990; Mintz, 2003). The ability to identify which of these mechanisms is most explanatory has been challenging. Indeed, many theories of early word learning take multiplicity of cue types and mechanisms as a central feature (e.g., Bloom, 2000; Hollich et al., 2000). As important as this work is, though, these studies are typically aimed at understanding how one or a small handful of words are learned in the laboratory under precisely defined learning conditions. They do not directly address questions regarding the developmental composition and ordering of growth in the lexicon across many different children in their natural environments, nor whether these patterns are consistent across different languages.

An alternate approach to word learning asks why some words are learned so early and some much later. This question about the order of the acquisition of first words can provide a different window into the nature of children’s language learning. Posed as a statistical problem, the challenge is to find what set of variables best predicts the age at which different words are acquired. Previous work using this approach has revealed that, in English, within a lexical category (e.g., nouns, verbs), words that are more frequent in speech to children are likely to be learned earlier (Goodman, Dale, & Li, 2008). Further studies have found evidence that a variety of other semantic and linguistic factors are related to word acquisition, such as salience and iconicity (Hills, Maouene, Maouene, Sheya, & Smith, 2009; Perry, Perlman, & Lupyan, 2015; Roy, Frank, DeCamp, Miller, & Roy, 2015; Stokes, 2010; Swingley & Humphrey, 2018).

But these exciting findings are limited in their generality because each study used a different dataset and focused on different predictors. In addition, nearly all studies to date have exclusively analyzed data from English-learning children, providing no opportunity for cross-linguistic comparison of the relative importance of the many relevant factors under consideration. Cross-linguistic comparisons are critical to identifying the universal mechanisms that are in play for all children and differentiating them from patterns of acquisition that emerge due to the particulars of a given language or culture (E. Bates & MacWhinney, 1987; Slobin, 1985). Our goal here is to extend these classic approaches by assessing the degree to which the predictors of word learning are consistent across different languages, as well as whether there are similar patterns across different lexical categories.

The primary tool for characterizing the breadth of children’s early vocabularies in these previous studies has been structured parent report. Naturalistic language samples and experimental methods are both valuable methods for assessing aspects of child language (Bornstein & Haynes, 1998; Fernald, Perfors, & Marchman, 2006). But outside of a few ultra-dense transcripts (e.g., Roy et al., 2015), neither method typically provides the kind of holistic and comprehensive view that comes from parent report. We focus in particular on the MacArthur-Bates Communicative Development Inventory (CDI; Fenson et al., 2007), a family of parent-report vocabulary checklists in which parents are asked whether their child “understands” or “understands and says” a large set of individual words.

The CDIs are an inexpensive and widely used method for gathering reliable and valid data about the nature and extent of young children’s productive and receptive vocabularies (see Fenson et al., 1994, for review; cf. Feldman et al., 2000; Fenson et al., 2000). Although CDIs cannot exhaustively capture all words in a child’s vocabulary (Mayor & Plunkett, 2011), they do give an estimate of a child’s knowledge about several hundred words, far more than the handful that are typically tested in a lab experiment. CDI estimates of vocabulary size are highly correlated with children’s vocabulary knowledge as assessed with naturalistic observation or using standardized tests (Fenson et al., 2007). Of course, any parent report measure is subject to reporting biases. The CDIs were designed to minimize these by asking parents to report only on observable behaviors that are currently (rather than retrospectively) demonstrated and to identify words from a preselected list (rather than having them recall them on their own).

Because of the low cost of administering CDI instruments, it is relatively easy to gather samples containing data about hundreds or thousands of children. Such large samples in turn make it possible to recover stable estimates of the average difficulty of individual words, even if individual children’s data may be noisy. Thus, CDI data are typically the dataset of choice for the studies of vocabulary composition described above.

Finally, CDI instruments have been adapted in dozens of different languages, providing an opportunity for cross-linguistic comparison. The American English CDI is not simply translated to other languages verbatim; instead, expert groups of researchers adapt the form for their particular linguistic and cultural situation. This process leads to a wide range of forms that share a common structure, but contain sets of words that are customized to a particular language and culture. Thus, cross-linguistic comparisons do not reflect children’s acquisition of a single set of words, but instead capture relevant information regarding patterns of children’s vocabulary development using instruments designed specifically for each language.^1^

In our study, we conduct cross-linguistic comparisons of the acquisition trajectories of children’s early-learned words using Wordbank (wordbank.stanford.edu; Frank, Braginsky, Yurovsky, & Marchman, 2016), an open repository that aggregates administrations of the CDI across languages. We integrate these acquisition trajectory data with independently derived characterizations of the word-learning environment from other datasets. The use of secondary datasets is warranted because no currently available resource provides data on both children’s language environments and their learning outcomes for more than a small handful of children. In particular, we derive our estimates of the language environment from transcripts of speech to children in the CHILDES database (MacWhinney, 2000) and measures of meaning-related word properties from available psycholinguistic databases. This data-integration methodology was originated by Goodman et al. (2008); it relies on large samples to average out the (substantial) differences among children and care environments. While introducing additional sources of variability, this approach allows for analyses that cannot be performed on smaller datasets that measure only children or environments but not both.

To measure environmental input, we used existing adult speech data from the CHILDES database to estimate each word’s frequency (a) in speech to children, (b) as a sole utterance constituent, (c) in utterance-final position, and the (d) mean length in words of utterances (MLU-w) containing that word. While crude, these measures are both easy to compute and relatively comparable across languages. To derive proxies for the meaning-based properties of each word, we accessed available psycholinguistic norms using adult ratings of each word’s (a) concreteness, (b) valence, (c) arousal, and (d) association with babies. Integrating these estimates, we predict each word’s acquisition trajectory, assessing the relative contributions of each predictor, how predictors change over development, and how predictors differ by lexical category. Since vocabulary composition differs in comprehension and production (e.g., Benedict, 1979), we conduct our analyses independently on each.

These analyses address two questions. First, we ask about the degree of consistency across languages in the relative importance of each predictor. To do so, we compare the estimates for the effect of each predictor for each language and conduct analyses that determine the likelihood that the consistency of the estimates did not occur by chance. Consistency in the patterning of predictors would suggest that similar information sources are important for learners, regardless of language, and that linguistic dissimilarities (e.g., greater morphological complexity in Russian, greater phonological complexity in Danish) do not dramatically alter the course of acquisition. Conversely, evidence for variability across languages would show the degree to which learners face different challenges in learning different languages, posing a challenge for more universalist accounts. Further, systematicity in the variability between languages would reveal which languages are more similar than others in the structure of these different challenges.

Second, we ask which lexical categories are most influenced by linguistic environment factors, like frequency and utterance length, compared with meaning-based factors like concreteness and valence. Division of dominance theory suggests that nouns might be more sensitive to meaning factors, while predicates and closed-class words might be more sensitive to linguistic environment factors (Gentner & Boroditsky, 2001). And on syntactic bootstrapping theories (Gleitman, 1990), nouns are argued to be learned via frequent co-occurrence (operationalized by frequency), while verbs might be more sensitive to syntactic factors (operationalized here by utterance length; Snedeker, Geren, & Shafto, 2007). Thus, examining the relative contribution of different predictors across lexical categories can help test the predictions of influential theories of acquisition.

## METHODS

The code and data for these analyses are available on GitHub (Braginsky, Yurovsky, Marchman, & Frank, 2019a).

### Acquisition Trajectories

To estimate the trajectory of words’ acquisition, we used vocabulary data collected using CDI instruments adapted in many different languages, including both Words & Gestures (WG) and Words & Sentences (WS) forms. When filling out a CDI form, parents are either asked to indicate whether their child “understands” (comprehension) or “understands and says” (production) each of around 400–700 words. Both comprehension and production are queried for younger children and only production is queried for older children. We included data from the items on the WG form for comprehension, and data from the items in common between the WG and WS forms for production. Placeholder items, such as “child’s own name,” were excluded. Table 1 gives an overview of our acquisition data (Acarlar et al., 2008; Bleses et al., 2008; Boudreault, Cabirol, Poulin-Dubois, Sutton, & Trudeau, 2007; Caselli et al., 1995; Caselli, Rinaldi, Stefanini, & Volterra, 2012; Eliseeva & Vershinina, 2009; Eriksson & Berglund, 2002; Jackson-Maldonado et al., 2003; Kovacevic, Babic, & Brozovic, 1996; Simonsen, Kristoffersen, Bleses, Wehberg, & Jørgensen, 2014; Trudeau & Sutton, 2011; Vershinina, Eliseeva, Lavrova, Ryskina, & Zeitlin, 2011; also see Supplemental Information Figure SI.1, Braginsky, Yurovsky, Marchman, & Frank, 2019b, for the age distributions). Each of the datasets was collected in the language of the community, e.g., the Mexican Spanish CDI data were collected in several areas of Mexico; longitudinal administrations were excluded.

**Table 1.** Statistics for data from Wordbank and CHILDES. N indicates number of children.

		Production		Comprehension		CHILDES	
Language	CDI items	N	Ages	N	Ages	Types	Tokens
Croatian	388	627	8−30	250	8−16	12,064	218,775
Danish	381	6,112	8−36	2,398	8−20	4,956	195,658
English (American)	393	7,312	8−30	1,792	8−18	45,597	7,679,042
French (Quebec)	396	1,364	8−30	537	8−16	28,819	2,551,113
Italian	392	1,400	7−36	648	7−24	7,544	188,879
Norwegian	380	7,466	8−36	2,374	8−20	10,670	231,763
Russian	410	1,805	8−36	768	8−18	5,191	32,398
Spanish (Mexican)	399	1,891	8−30	788	8−18	33,529	1,609,614
Swedish	371	1,367	8−28	467	8−16	8,815	359,155
Turkish	395	3,537	8−36	1,115	8−16	6,503	44,347

*Note*. CDI = MacArthur-Bates Communicative Development Inventory.

**Figure 1.**
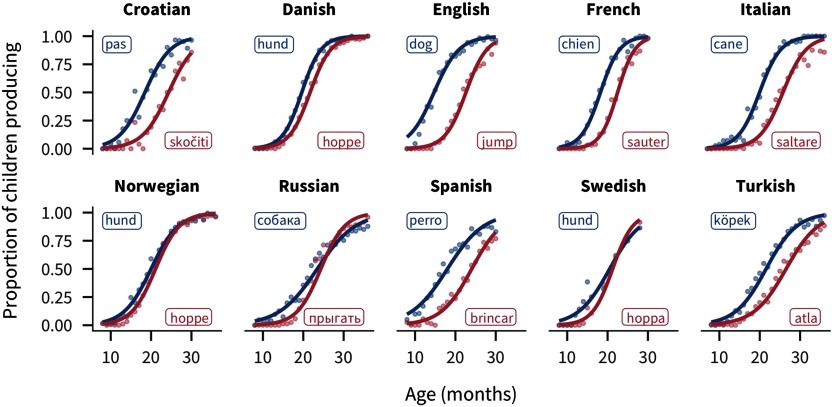
**Exampleproductiontrajectoriesforthewords“dog”and“jump” across languages.** Points show the proportion of children producing each word for each one-month age group. Lines show the best-fitting logistic curve. Labels show the forms of the words in each language.

For each word, the CDI data yield a trajectory reflecting the number of children that are reported to understand or produce the word at each age covered by the instrument (see Figure 1 for some examples).

### Word Properties

#### Overview.

For each word in each of our 10 languages, we used corpora of child-directed speech in that language from CHILDES to obtain an estimate of its frequency, the mean length of utterances in which it appears, its frequency as the sole constituent of utterance, and its frequency in utterance final position. We also computed each word’s length in phonemes.

In addition, each word’s concreteness, valence, arousal, and relatedness to babies^2^ were compiled from ratings based on previous studies using adult raters. Since existing ratings are primarily available for English, we mapped all words onto translation equivalents across CDI forms, verified by native speaker judgment, allowing us to use the English ratings across languages. Of the resulting translation equivalent meanings, 35% occur only in one language, 51% occur in more than one but not all languages, and 14% occur in all languages. While necessarily imperfect, this method allows us to examine languages for which limited resources exist. Example words for these predictors in English are shown in Table 2 (also see Figures SI.2 and SI.3, Braginsky et al., 2019b, for the distributions of values of each predictor).

**Table 2.** Items with the highest and lowest values for each predictor in English.

Predictor	Highest	Lowest
Arousal	naughty, money, scared	today, asleep, shh
Babiness	baby, bib, bottle	jeans, penny, donkey
Concreteness	apple, baby, ball	that, now, how
Final frequency	book, it, there	put, when, give
Frequency	you, it, that	babysitter, rocking chair, grrr
MLU-w	daddy, when, day	ouch, thank you, peekaboo
Number phonemes	refrigerator, cockadoodledoo, babysitter	i, eye, ear
Solo frequency	no, yes, thank you	feed, bathroom, tooth
Valence	happy, hug, love	ouch, hurt, sick

*Note*. MLU-w = mean length of utterances.

Each numeric predictor was centered and scaled (within language) so that all predictors would have comparable units.

#### Frequency.

For each language, we derived unigram counts based on adult speech in all corpora in CHILDES for that language. Frequencies varied widely both within and across lexical categories (see Figure SI.4, Braginsky et al., 2019b). Each word’s count was summed across inflected forms (e.g., “dogs” counts as “dog”) and synonyms (e.g., “father” counts as “daddy”). For polysemous words (e.g., “orange” as in color or fruit), occurrences were split uniformly between the senses on the CDI (there were only between 1 and 10 such word pairs in the various languages; in the absence of cross-linguistic corpus resources for sense disambiguation, this is a necessary simplification). Counts were normalized to the length of each corpus, Laplace smoothed (i.e., counts of 0 were replaced with counts of 1), and log transformed.

#### Solo and Final Frequencies.

Using the same dataset as for frequency, we estimated the frequency with which each word occurred as the sole word in an utterance, and the final word of an utterance (not counting single-word utterances). Solo and final counts were normalized to the length of each corpus, Laplace smoothed, and log transformed. Since both of these estimates are by necessity highly correlated with frequency, we then residualized unigram frequency out of both, so that values reflect an estimate of the effects of solo frequency and final frequency over and above frequency.

#### MLU-w.

MLU-w is only a rough proxy for syntactic complexity, but is relatively straightforward to compute across languages (in contrast to other metrics). For each language, we estimated each word’s MLU-w by calculating the mean length in words of the utterances in which that word appeared, for all corpora for that language. For words that occurred fewer than 10 times, MLU-w estimates were treated as missing.

#### Number of Phonemes.

In the absence of consistent resources for cross-linguistic pronunciation, we computed the number of phonemes in each word in each language based on phonemic transcriptions of each word obtained using the eSpeak tool (Duddington, 2012). We then spot-checked these transcriptions for accuracy.

#### Concreteness.

We used previously collected norms for concreteness (Brysbaert, Warriner, & Kuperman, 2014), which were gathered by asking adult participants to rate how concrete the meaning of each word is on a 5-point scale from abstract to concrete.

#### Valence and Arousal.

We also used previously collected norms for valence and arousal (Warriner, Kuperman, & Brysbaert, 2013), for which adult participants were asked to rate words on a 1–9 happy-unhappy scale (valence) and 1–9 excited-calm scale (arousal).

#### Babiness.

We used previously collected norms of “babiness,” a measure of association with infancy (Perry et al., 2015) for which adult participants were asked to judge a word’s association with babies on a 1–10 scale.

#### Lexical Category.

Category was determined on the basis of the conceptual categories presented on the CDI form (e.g., “Animals,” “Action Words”), such that the Nouns category contains common nouns, Predicates contains verbs, adjectives, and adverbs, Function Words contains closed-class words (following E. Bates et al., 1994), and the remaining items are binned as Other.

#### Imputation.

The resulting set of predictor value for each language had varying numbers of missing values, depending on resource availability (number phonemes 0%, concreteness 0%–1%, arousal and valence 8%–13%, [solo/final] frequency 2%–14%, babiness 10%–33%, MLU-w 2%–53%). We used iterative regression imputation within each language to fill in these missing values by first replacing missing values with samples drawn randomly with replacement from the observed values, and then iteratively imputing values for each predictor based on a linear regression fitting that predictor from all others.

#### Collinearity.

A potential concern for comparing coefficient estimates is predictor collinearity. Fortunately, in every language, the only relatively high correlations were between MLU-w and solo frequency (mean over languages *r* = −0.44), as expected given the similarity of these factors, along with modest correlations between frequency and concreteness (mean over languages *r* = −0.36) and between frequency and number of phonemes (mean over languages *r* = −0.33), a reflection of Zipf’s Law (Zipf, 1935). More importantly, the variance inflation factor for each predictor in each language was no greater than 2.27, indicating that multicollinearity among the predictors is low (see Figure SI.5 for the full set of pairwise correlations and Figure SI.6 for the variance inflation factors, Braginsky et al., 2019b).

### Analysis

We used mixed-effects logistic regression models (fit with the MixedModels package in Julia; D. Bates et al., 2018) to predict whether each child understands/produces each word from the child’s age, properties of the word, interactions between each property and age, and interactions between each property and lexical category (which was contrast coded). Each model was fit to all data from a particular language and included a random intercept for each word and a random slope of age for each word. Computational and technical limitations prevented us from including random effects for child or including data from all languages in one joint model.

The magnitude of the standardized coefficient on each property gives an estimate of its independent contribution to words being understood/produced by more children. Interactions between properties and age give estimates of how this effect is modulated for earlier-learned and later-learned words. For example, a positive effect of babiness means that words associated with babies are learned earlier; a negative interaction with age means that high babiness primarily leads to higher rates of production and comprehension for younger children. Similarly, interactions between properties and lexical category give estimates of how the effect differs among nouns, predicates, and function words.

## RESULTS

### English Predictor Effects

**Figure 2.**
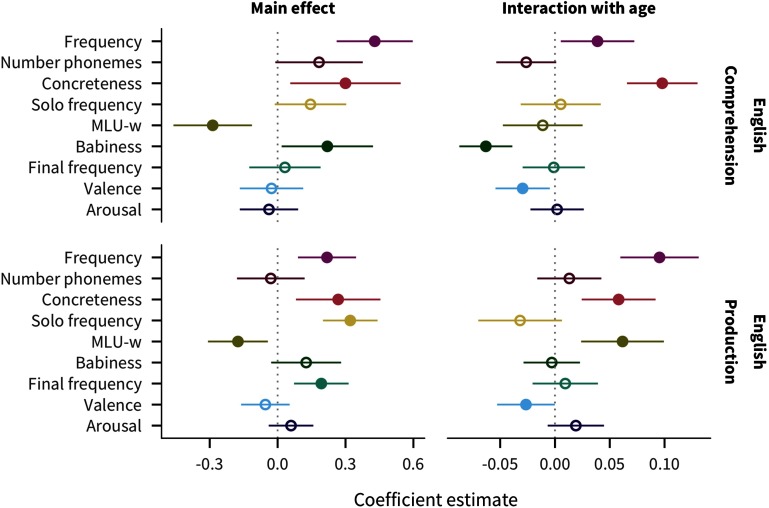
**Estimates of coefficients in predicting words’ developmental trajectories for English comprehension and production data.** Larger coefficient values indicate a greater effect of the predictor on acquisition: positive main effects indicate that words with higher values of the predictor tend to be understood/produced by more children, while negative main effects indicate that words with lower values of the predictor tend to be understood/produced by more children; positive age interactions indicate that the predictor’s effect increases with age, while negative age interactions indicate the predictor’s effect decreases with age. Line ranges indicate 95% confidence intervals; filled in points indicate coefficients for which *p* < .05.

To illustrate the structure of our analysis, we first describe the results for English data, shown in Figure 2 as the main effect and age interaction coefficient estimates and 95% confidence intervals, for comprehension and production. For main effects, words are more likely to be known by more children if they are higher in frequency or concreteness, as well as in babiness for comprehension and in sentence-final frequency or sole-constituent frequency for production. In contrast, words that appear in shorter sentences (MLU-w) are more likely to be reported as understood or produced. For age interactions, while most predictors have consistent effects over age, words that are higher in frequency or concreteness are more likely to be known more by older children, while words that are higher in valence have a greater effect on acquisition in younger children, with an additional negative interaction with babiness in comprehension and positive interaction with MLU-w in production.

### Cross-linguistic Predictor Effects

**Figure 3.**
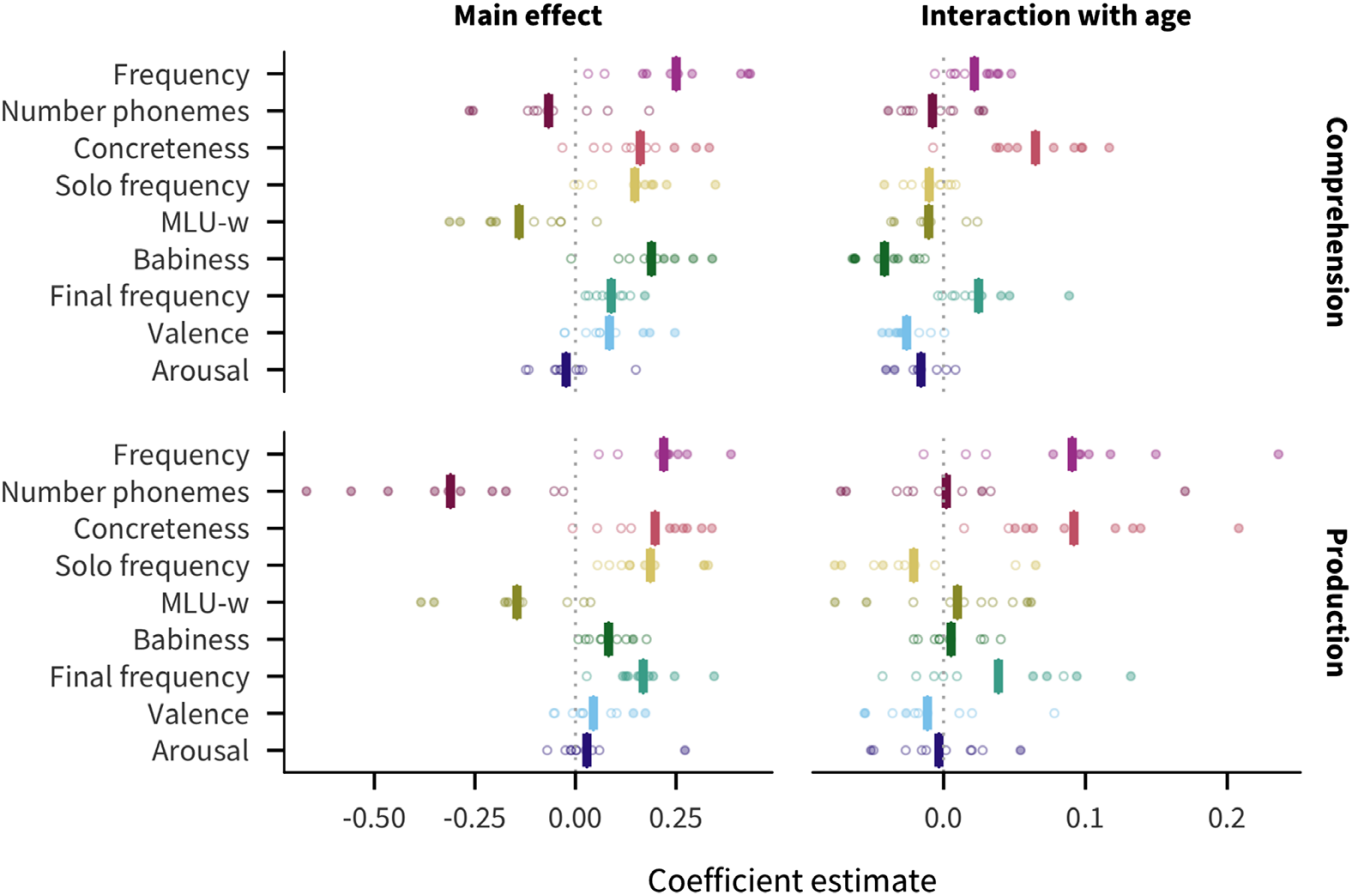
**Estimates of coefficients in predicting words’ developmental trajectories for all languages and measures.** Each point represents a predictor’s coefficient in one language, with the bar showing the mean across languages. Filled in points indicate coefficients for which *p* < .05.

Figure 3 shows the coefficient estimate for each predictor in each language and measure (for additional visualizations of the coefficients, see Figures SI.7, SI.8, and SI.9, Braginsky et al., 2019b). We find that frequency is the strongest predictor of acquisition (mean across languages and measures $\bar{\beta }$ = 0.23). Other relatively strong overall predictors include concreteness ($\bar{\beta }$ = 0.18), solo frequency ($\bar{\beta }$ = 0.17), MLU-w ($\bar{\beta }$ = −0.14), and final frequency ($\bar{\beta }$ = 0.13). Number of phonemes is comparatively large for production ($\bar{\beta }$ = −0.31) but not comprehension ($\bar{\beta }$ = −0.07); conversely, babiness is comparatively large for comprehension ($\bar{\beta }$ = 0.19) but not production ($\bar{\beta }$ = 0.08). Finally, valence ($\bar{\beta }$ = 0.06) and arousal ($\bar{\beta }$ = 0.003) have much smaller effects.

Given the emphasis on frequency effects in the literature (Ambridge, Kidd, Rowland, & Theakston, 2015), one might have expected frequency to dominate, but several other predictors are also quite strong. In addition, some factors previously argued to be important for word learning, namely valence and arousal (Moors et al., 2013), appear to have limited relevance when compared to other factors. These results provide a strong argument for our approach of including multiple predictors and languages in our analysis.

### Consistency

Apart from valence and arousal, all other predictors have the same the direction of effect in all or almost all languages and measures (at least 17 of the 20). Thus, across languages, words are likely to be understood and produced by more children if they are more frequent, shorter, more concrete, more frequently the only word in an utterance, more associated with babies, more frequently the final word in an utterance, and appear in shorter utterances.

**Figure 4.**
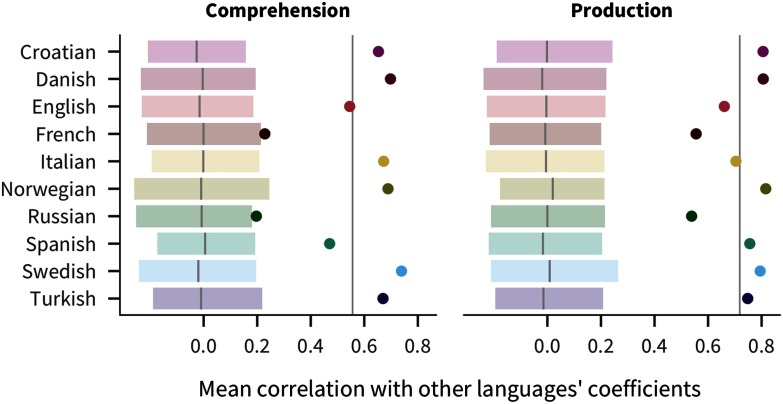
**Correlations of coefficient estimates between languages.** Eachpoint represents the mean of one language’s coefficients’ correlation with each other language’s coefficients, with the vertical line indicating the overall mean across languages. The shaded region and line show a bootstrapped 95% confidence interval for a randomized baseline where predictor coefficients are shuffled within language.

Additionally, there is considerable consistency in the magnitudes of predictors across languages. A priori it could have been the case that different languages have wildly different effects of various factors (due to linguistic or cultural differences), but this pattern is not what we observe. Instead, there is more consistency in the correlations between coefficients across languages than would be expected by chance. As shown in Figure 4, each language’s mean pairwise correlation with other languages’ coefficients (i.e., the correlation of coefficients for English with coefficients for Russian, for Spanish, and so on) is outside of bootstrapped estimates in a randomized baseline created by shuffling predictor coefficients within language. The pairwise correlations are more consistent for production (mean 0.72) than for comprehension (mean 0.56), in which French and Russian effects are more idiosyncratic.

### Variability

While some particular coefficients differ substantially from the trend across languages (e.g., the effect of frequency for comprehension in Spanish is near 0), these individual datapoints are difficult to interpret. Many unmeasurable factors could potentially account for these differences: Spanish frequency estimates could be less accurate due to corpus sparsity or idiosyncrasy, the samples of children in the Spanish CDI or CHILDES data could differ more demographically, or Spanish-learning children could in fact rely less on frequency. Rather than attempting to interpret individual coefficients, we instead ask how the patterns of difference among languages reflect systematic substructure in the variability of the effects.

**Figure 5.**
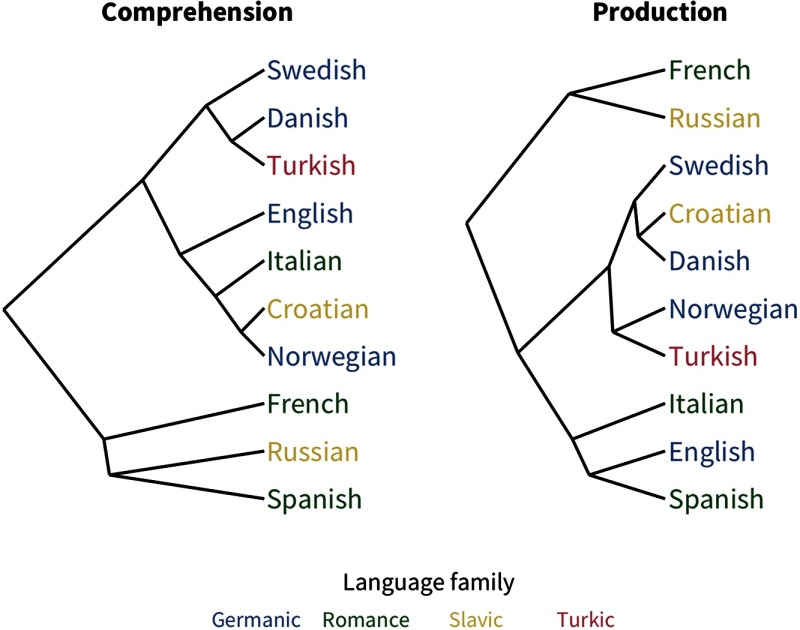
**Dendrograms of the similarity structure among languages’ coefficients.**

To examine the substructure of predictor variability, we used hierarchical clustering analysis to find the similarity structure among the pairwise correlations between languages’ predictors. The resulting dendrograms are shown in Figure 5; these broadly reflect language typology, especially for production data. This result suggests that some language-to-language similarity is captured by the profile of coefficient magnitudes our analysis returns.

### Comprehension vs. Production

As mentioned above, word length is the one predictor of acquisition that varied substantially between measures: it is far more predictive for production than comprehension. Thus, as measured here, length seems to reflect effects of production constraints (i.e., how difficult a word is to say) rather than comprehension constraints (i.e., how difficult it is to store or access). This result may explain why the hierarchical clustering analysis above appears more similar to linguistic typology in production than comprehension, that is, the role of production difficulty may be more similar for more typologically related languages. Another possibility is that since the measures are confounded with age (comprehension is only measured for younger children), word length may play a larger role later in acquisition. Similarly, the stronger effect of babiness in comprehension over production could be due to its larger prominence earlier in development.

### Developmental Change

For both comprehension and production, positive age interactions can be seen in at least 9 out of 10 languages for concreteness and frequency. Conversely, there are negative age interactions for babiness and valence for comprehension in at least 9 out of 10 languages. This suggests that concreteness and frequency facilitate learning more so later in development, while babiness and valence facilitate learning earlier in development. This result is consistent with the speculation above that the babiness predictor captures meanings that have special salience to very young infants.

### Lexical Categories

**Figure 6.**
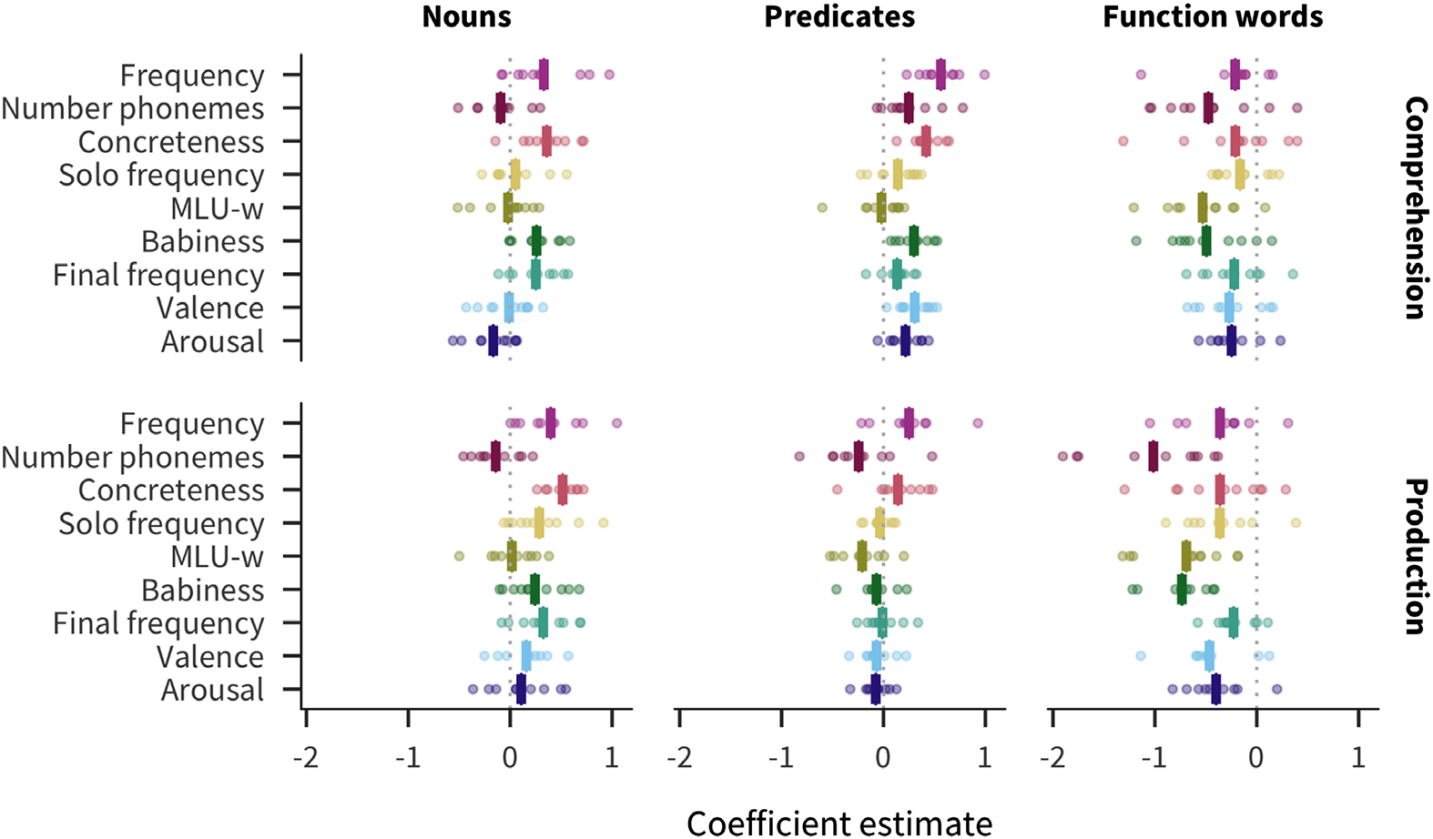
**Estimates of effects in predicting words’ developmental trajectories for each language, measure, and lexical category (main effect of predictor + main effect of lexical category + interaction between predictor and lexical category).** Each point represents a predictor’s effect in one language, with the bar showing the mean across languages.

Previous work suggests that predictors’ relationship with age of acquisition differs among lexical categories (Goodman et al., 2008). We investigate these differences by including lexical category interaction terms in our model. Figure 6 shows the resulting effects for each lexical category, combining the main effect of a given predictor with the main effect of the lexical category and the interaction between that predictor and that lexical category (see also Figures SI.10 and SI.11, Braginsky et al., 2019b).

Across languages, the strongest predictors of acquisition for both nouns and predicates are concreteness (nouns $\bar{\beta }$ = 0.44; predicates $\bar{\beta }$ = 0.28) and frequency (nouns $\bar{\beta }$ = 0.36; predicates $\bar{\beta }$ = 0.41). Thus content words are most likely to be known by more children if they are more frequent or more concrete. Conversely, function words are most influenced by number of phonemes ($\bar{\beta }$ = −0.74), babiness ($\bar{\beta }$ = −0.61), and MLU-w ($\bar{\beta }$ = −0.61), meaning that function words are most likely to be known by more children if they are shorter, less associated with babies, or appear in shorter sentences. These patterns are supportive of the hypothesis that different word classes are learned in different ways, or at least that the bottleneck on learning tends to be different, leading to different information sources being more or less important across categories.

Additionally, the mean pairwise correlation of coefficients between languages is much larger for nouns (0.68) and predicates (0.54) than for function words (0.29). The higher between-language variability for function words suggests the learning processes differ substantially more across languages for function words than they do for content words (see Figure SI.12, Braginsky et al., 2019b).

## DISCUSSION

What makes words easier or harder for young children to learn? Previous experimental work has largely addressed this question using small-scale laboratory studies. While such experiments can identify sources of variation, they typically do not allow for different sources to be compared directly. In contrast, observational studies allow the effects of individual factors to be measured across ages and lexical categories (e.g., Goodman et al., 2008; Hills et al., 2009; Swingley & Humphrey, 2018), but are limited in the size and scope of the datasets and languages that can be directly compared. The current analyses take advantage of recent innovative approaches via Wordbank, a large, cross-linguistic dataset of parent report instruments. By compiling data regarding early lexical development across 10 languages and examining patterns of acquisition in relation to 9 predictors, our work expands the scope of these studies dramatically, leading to several new findings.

First, we found consistency in the patterning of predictors across languages at a level substantially greater than the predictions of a chance model. This consistency supports the idea that differences in culture or language structure do not lead to fundamentally different acquisition strategies, at least at the level of detail we were able to examine. Instead, they are likely produced by processes that are similar across populations and languages. Such processes could include learning mechanisms or biases internal to children, or interactional dynamics between children or caregivers. We believe these consistencies should be an important topic for future investigation.

Second, predictors varied substantially in their weights across lexical categories. Frequent, concrete nouns were learned earlier, consistent with theories that emphasize the importance of early referential speech (e.g., Baldwin, 1995). For predicates, concreteness was somewhat less important and frequency was somewhat more important. And for function words, length and MLU-w were more predictive, perhaps because it is easiest to decode the meanings of function words that are used in short sentences (or because such words have meanings that are easiest to decode). Overall, these findings are consistent with some predictions of both division of dominance theory, which highlights the role of conceptual structure in noun acquisition (Gentner & Boroditsky, 2001), and syntactic bootstrapping theory, which emphasizes linguistic structure over conceptual complexity in the acquisition of lexical categories other than nouns (Snedeker et al., 2007). More generally, our methods here provide a way forward for testing the predictions of these theories across languages and at the level of the entire lexicon rather than individual words.

In addition to these new insights, several findings emerged that confirm and expand previous reports. Environmental frequency was an important predictor of learning, with more frequently heard words learned earlier (Goodman et al., 2008; Swingley & Humphrey, 2018). Predictors also changed in relative importance across development. For example, certain words whose meanings were more strongly associated with babies appeared to be learned early for children across the languages in our sample (as in Tardif et al., 2008). Finally, word length showed a dissociation between comprehension and production, suggesting that challenges in production do not carry over to comprehension (at least in parent-report data).

Despite its larger scope, our work shares a number of important limitations with previous studies. First and foremost, our approach is to predict acquisition data for one set of individuals from data about the experiences of a completely different set of individuals and from conceptual ratings gathered from yet others. In contrast to dense-data analyses (Roy et al., 2015), this approach fundamentally limits the amount of variability we will be able to capture. Second, the granularity of the predictors that can be extracted from corpus data and applied to every word is necessarily quite coarse. Ideally, predictors could be targeted more specifically at particular theoretical constructs of interest (e.g., the patterns of use for specific predicates). Third, our analyses are conducted within language, so to the extent that the predictors can have differing ranges in different languages, cross-linguistic patterns in predictor effects could be obscured.

Finally, our data are observations gleaned from parent report. CDI instruments are both reliable and valid, and the cross-linguistic adaptations we used contain the original researchers’ best attempts to create culturally appropriate word lists. Nevertheless, this observational design introduces many sources of uncertainty and bias. First, the open data format of Wordbank reflects the sampling and administration methods of many groups around the world; these introduce many unknown biases that we cannot control (though they would likely not contribute to observed consistencies). Second, language and culture covary completely in our sample and so variability that we observe cannot be attributed to one or the other. Finally, some observed consistencies could arise from consistency in parental reporting biases. For example, across languages, parents might be generally biased to underreport comprehension of function words. Despite the quantity of data analyzed here, our conclusions will require further testing through converging evidence from both laboratory experiments and direct observation.

In sum, by examining predictors of early word learning across languages, we identified substantial cross-linguistic consistency in the factors contributing to the ease or difficulty of learning individual words. This suggests that common learning mechanisms and/or environmental supports for learning are shared across all of these languages. These findings also testify to the importance of building open, shared resources in the study of child language learning—without the efforts of many research groups across many language communities, studies like ours would be impossible. Additionally, we hope that our work here provides a baseline for the building of future predictive models that allow theories of language learning to be tested at scale.

## ACKNOWLEDGMENTS

Thank you to the labs and individuals who contributed data to Wordbank.

## FUNDING INFORMATION

National Science Foundation, Award ID 1528526 (to MCF); Zhou Fund for Language and Cognition Research, Stanford University (to MCF); Jacobs Foundation Research Fellowship (to MCF); National Institutes of Health National Research Service Award (to DY); James S. McDonnell Scholar Award (to DY).

## AUTHOR CONTRIBUTIONS

MB: Conceptualization: Equal; Data curation: Lead; Formal analysis: Lead; Funding acquisition: Supporting; Methodology: Equal; Visualization: Lead; Writing - Original Draft: Lead; Writing - Review & Editing: Equal. DY: Conceptualization: Equal; Data curation: Equal; Formal analysis: Equal; Funding acquisition: Supporting; Methodology: Equal; Visualization: Supporting; Writing - Original Draft: Supporting; Writing - Review & Editing: Supporting. VAM: Conceptualization: Equal; Data curation: Supporting; Formal analysis: Equal; Funding acquisition: Equal; Methodology: Equal; Visualization: Supporting; Writing - Original Draft: Equal; Writing - Review & Editing: Equal. MCF: Conceptualization: Lead; Data curation: Supporting; Formal analysis: Equal; Funding acquisition: Lead; Methodology: Equal; Visualization: Supporting; Writing - Original Draft: Equal; Writing - Review & Editing: Lead.

## Notes

^1^ Of course, observational data of this type are still open to other sources of bias, a point we return to in the Discussion.^2^ Previous studies have shown robust consistency in the types of words that children learn very early (Tardif et al., 2008). These words seem to describe concepts that are important or exciting in the lives of infants in a way that standard psycholinguistic features like concreteness do not. Capturing this intuition quantitatively is difficult, but Perry et al. (2015) provide a proxy measure as a first step. This measure is simply the degree to which a particular word was “associated with babies.” Intuitively, we expect this measure to capture the degree to which words like “ball” or “bottle” feature heavily in the environment (and presumably, mental life) of many babies.
