# Supplementary material for: Consistency and Variability in Children’s Word Learning Across Languages
Source: Open Mind (Camb). 2019 Jun 1;3:52–67. doi: 10.1162/opmi_a_00026 (PMC6716390; doi:10.1162/opmi_a_00026)
Supplement: Supplementary file 1 [file opmi-03-52-s001.pdf]

SUPPLEMENTAL INFORMATION

In this supplemental information document, we include a variety of visualizations that provide additional information about our datasets and models. As noted in the manuscript, all of the code and data for our analyses are available at github.com/mikabr/aoa-prediction. In addition to the code for wrangling and analyzing all the data, this includes cached versions of the all the intermediate results, such as all of the coefficient estimates. We welcome extensions of our work or alternate analyses of our data – feel free to contact the corresponding author at mikabr@mit.edu with any questions.

Model specification

All models were fit in Julia using the MixedModels package. For a given subset of the data (e.g. data for English production), for each CDI item (`item`), we computed the proportion of children reported to understand/produce it (`prop`) and the total number of children (`total`). We then fit a generalized linear mixed model to the data subset, with a binomial response distribution, the values in `total` as the trial weights, and the following formula:

```
prop ~ (age | item) + age * arousal + age * babiness + age * concreteness
       + age * final_frequency + age * frequency + age * MLU
       + age * num_phons + age * solo_frequency + age * valence
       + lexical_category * arousal + lexical_category * babiness
       + lexical_category * concreteness + lexical_category * final_frequency
       + lexical_category * frequency + lexical_category * MLU
       + lexical_category * num_phons + lexical_category * solo_frequency
       + lexical_category * valence
```

So the regression predicts the proportion of successes (proportion of children who understand/produce each item) out of the total number of trials (number of children) from the children's age, each item-level predictor, the interactions between age and each item-level predictor, and the interactions between lexical category and each item-level predictor, with a random slope for age by item.

Age distributions

We report the distribution of children's ages for each language and measure. This addresses the potential concern that large differences in age between samples could unbalance the design. The age distributions largely overlap, which makes sense given that many of the datasets are from normative studies that attempted to sample evenly across ages.

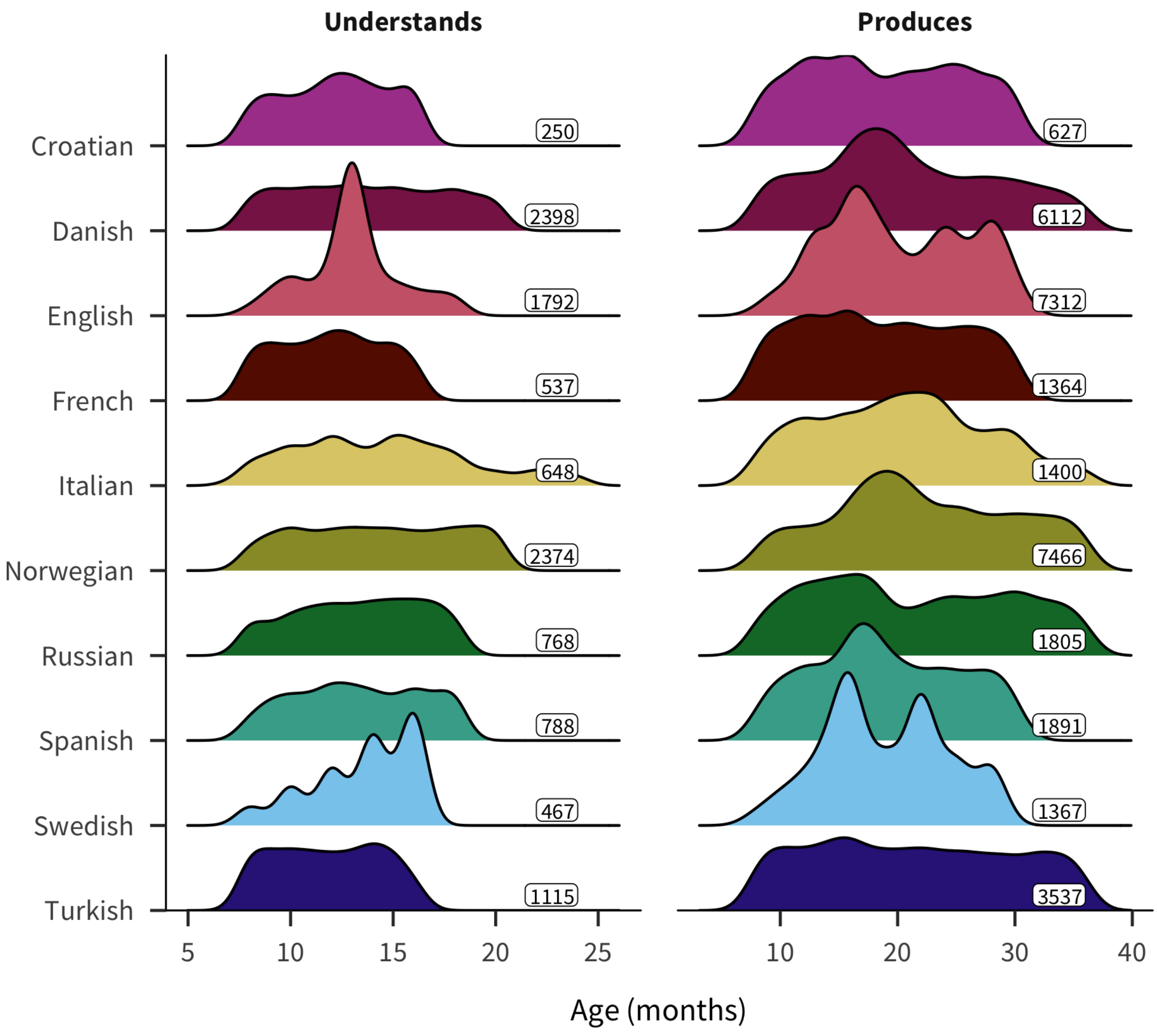

Figure SI.1. Densities of children's ages for each language and measure, along with a label for the total numbers of children.

Predictor values

As another illustration of the structure of our dataset, we show the distributions of the values of each predictor in each language, first for the raw values and then for the imputed, centered, and scaled values.

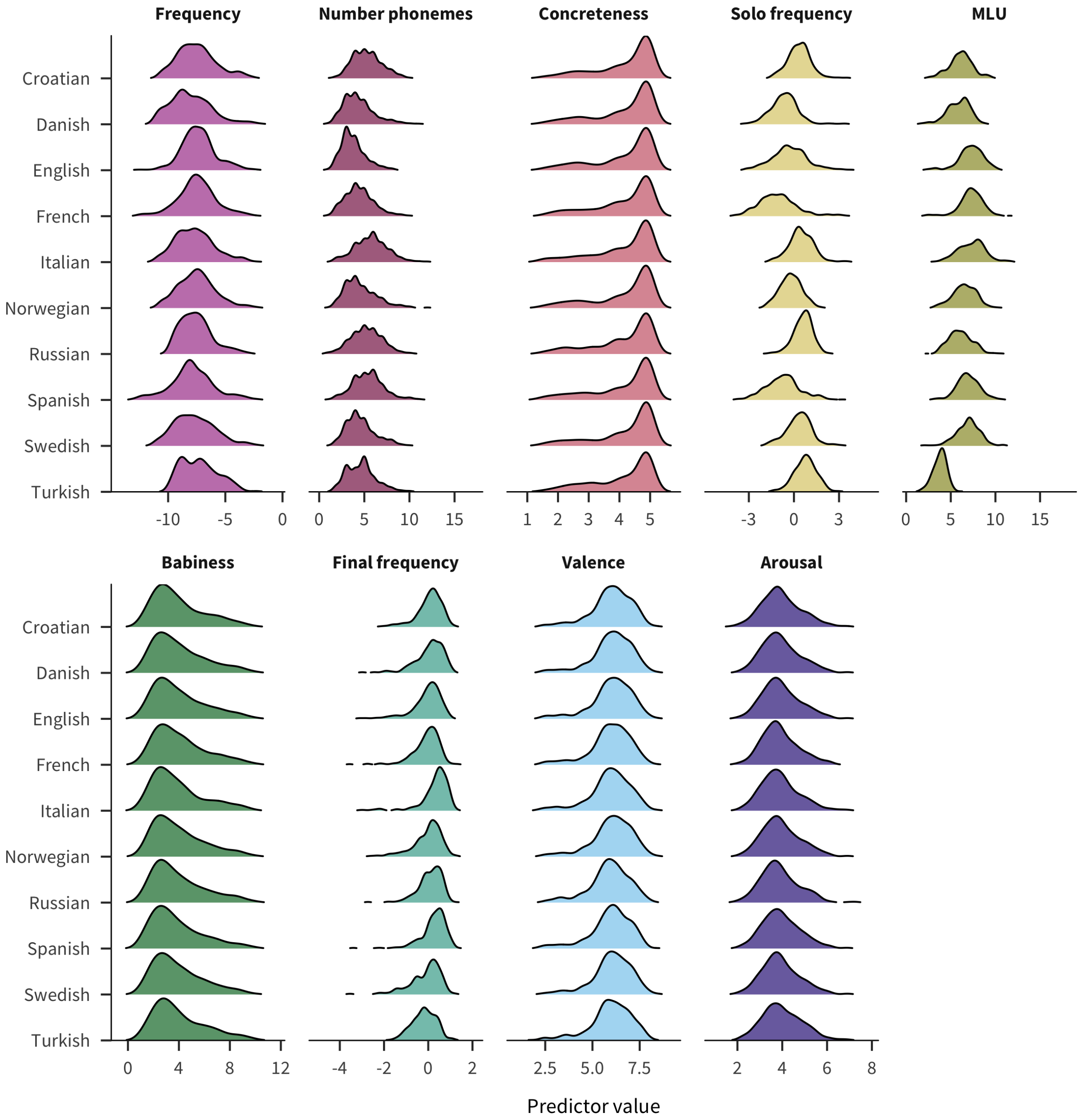

Figure SI.2. Densities of the raw values of each predictor for each language.

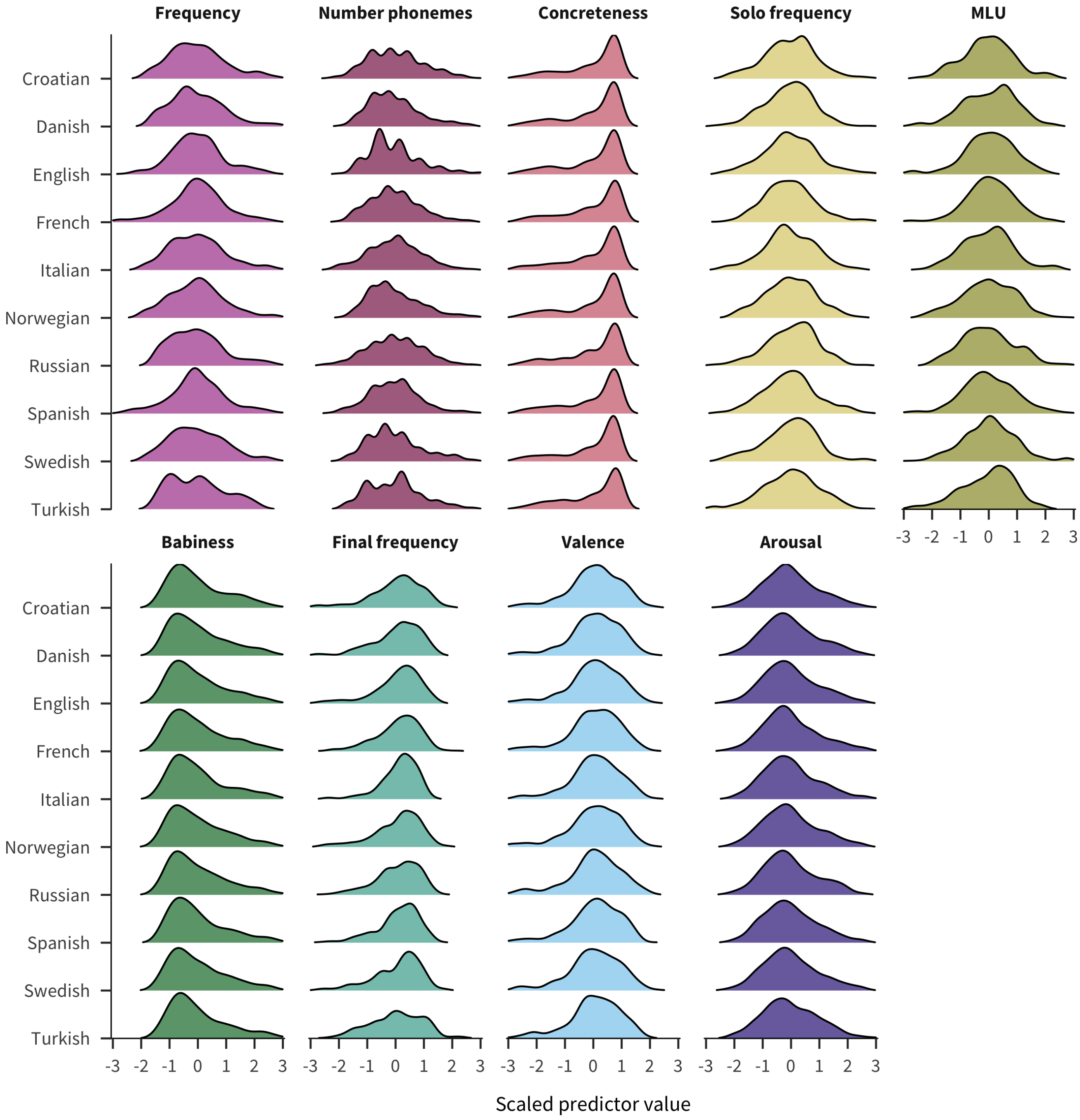

Figure SI.3. Densities of the imputed, centered, and scaled values of each predictor for each language.

Frequency and lexical category

A potential concern about our lexical category analysis is that lexical category might not be dissociable from frequency, in the sense that closed-class words are more frequent that open-class words and span a more narrower range of frequencies. The distribution of frequencies by lexical category in our dataset, shown below, demonstrates that there is substantial overlap in frequency between the closed-class and open-class words.

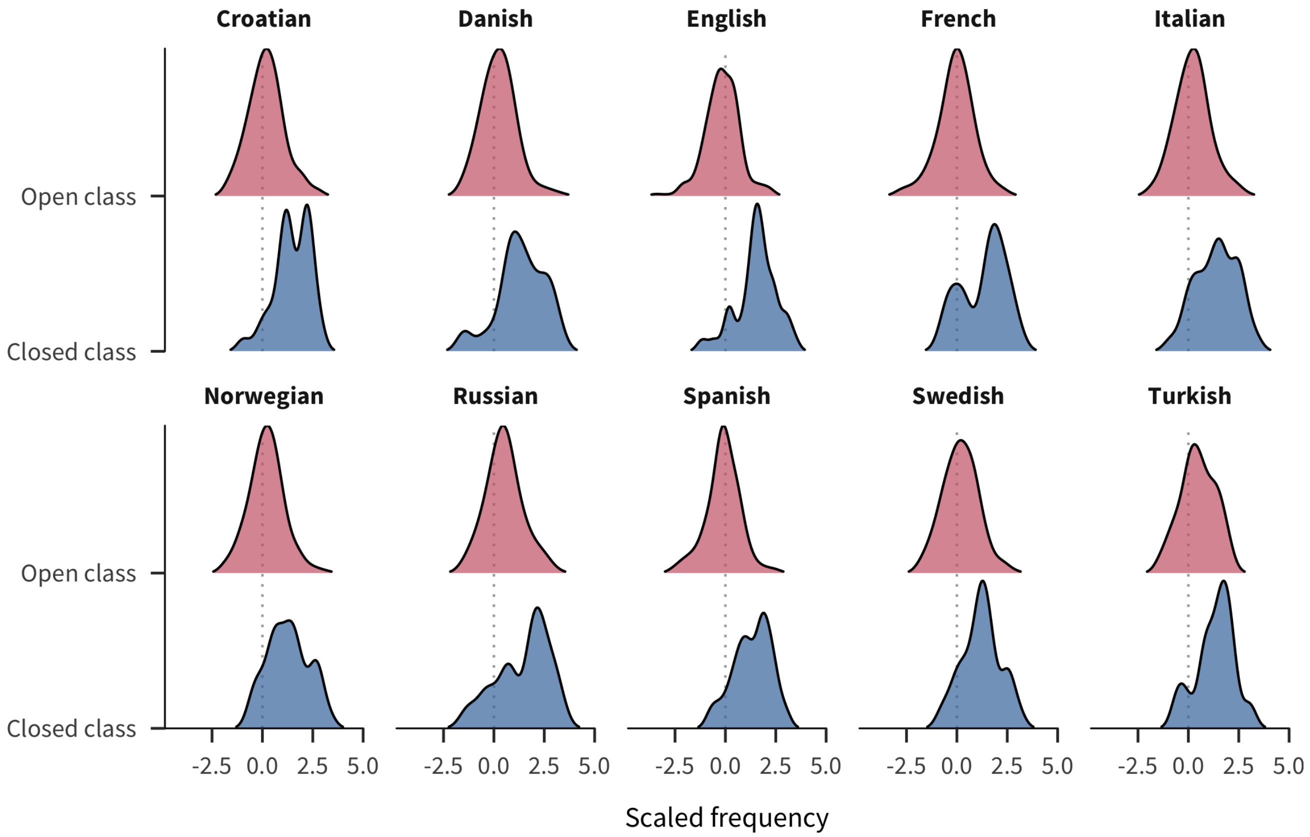

Figure SI.4. Densities of the frequency estimates for open and closed class words in each language.

Pairwise predictor correlations

In addition to the univariate distribution of each predictor shown above, we show the correlation between each pair of predictors. Too many large correlations between predictors could limit the interpretability of our coefficient estimates.

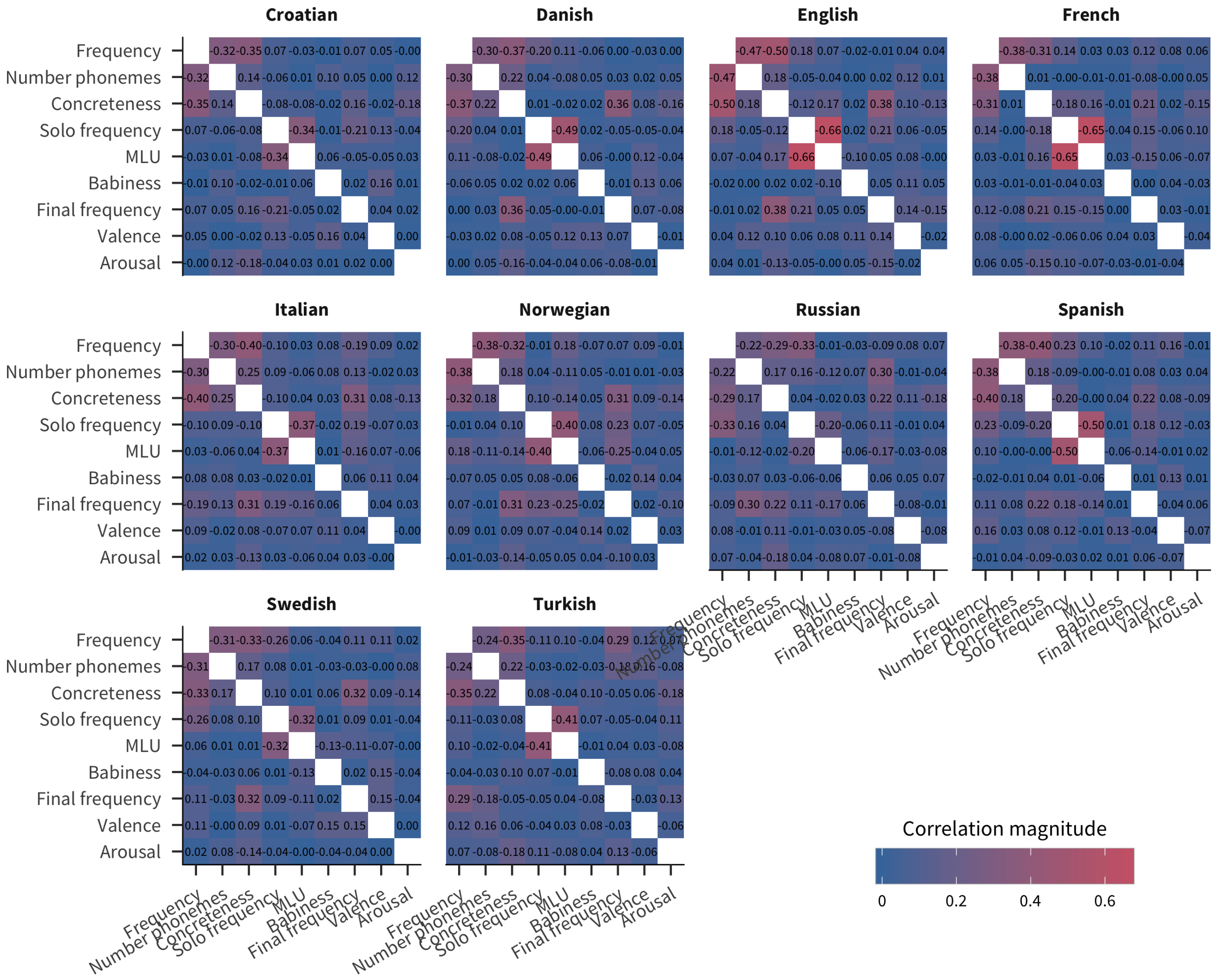

Figure SI.5. Pairwise correlations between predictors.

Variance inflation factors

To address the potential issue of multicollinearity in our models, we show the variance inflation factor (VIF) for each predictor in each language. VIF is computed for a predictor by fitting an ordinary least squares regression with that predictor as the dependent variable and all other predictors as the independent variables, getting its R^2, and then computing VIF $= \frac{1}{1-R^2}$.

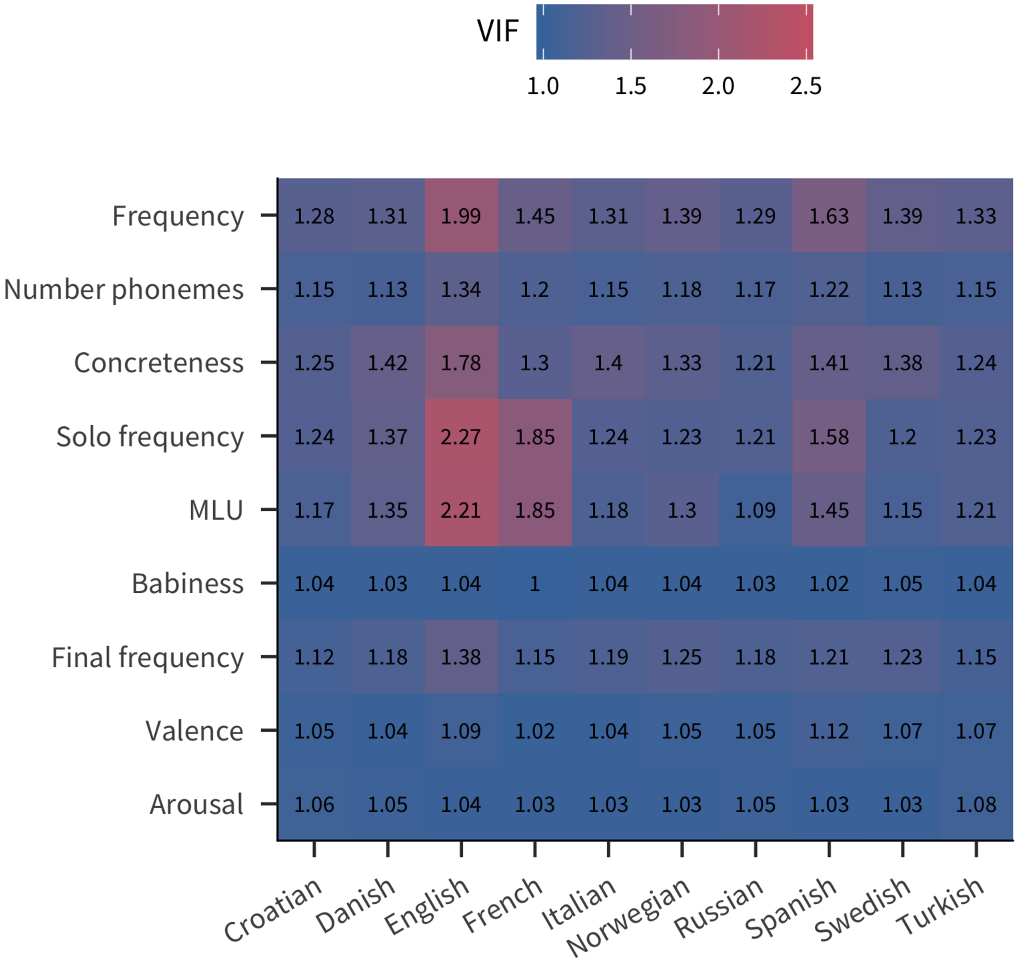

Figure SI.6. Variance inflation factors of the predictors.

Coefficients by language

As a supplement to Figure 3, we show the coefficient estimates for each language, first for the main effects and then for the age interactions.

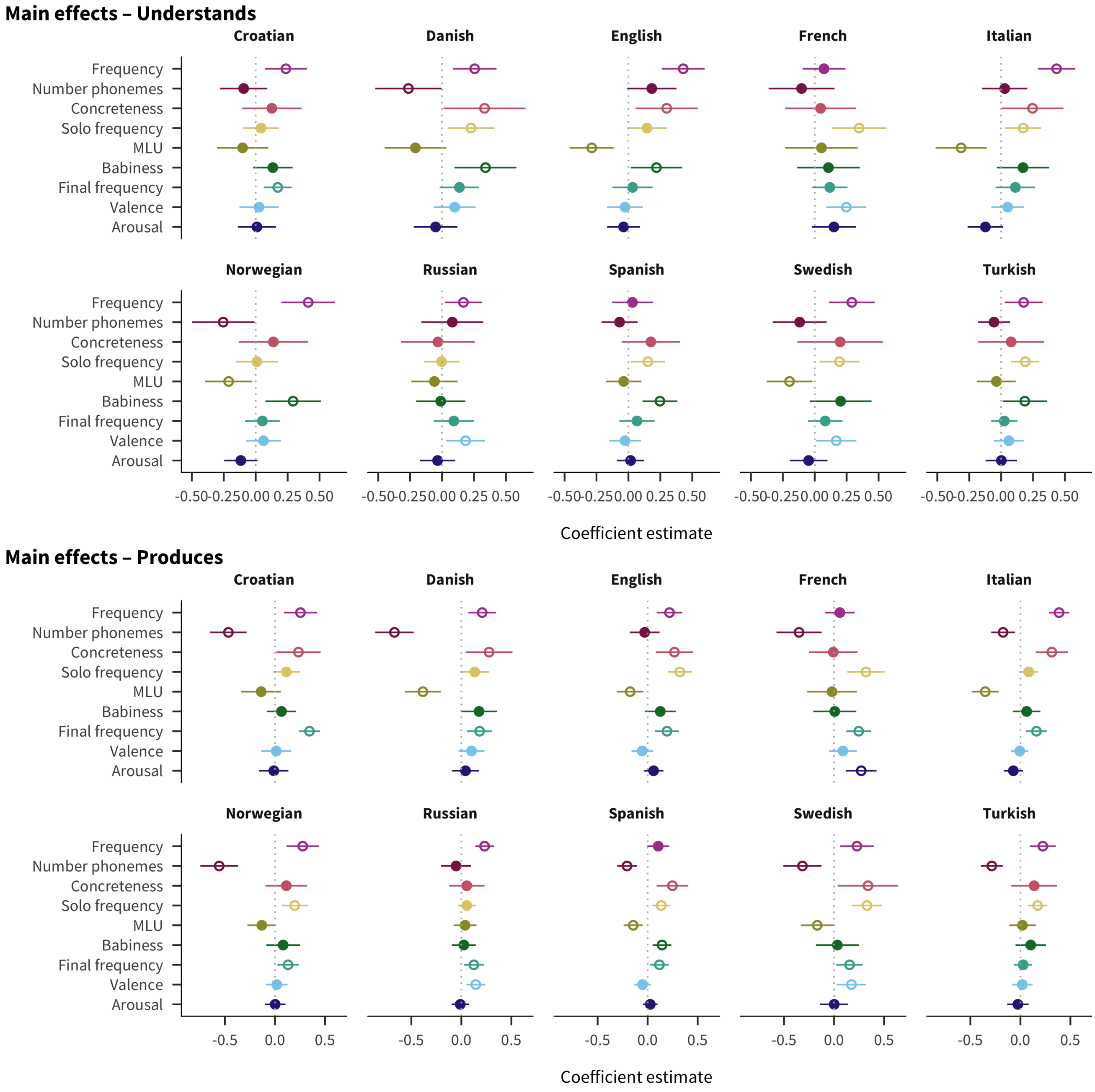

Figure SI.7. Main effects for each language and measure with 95% confidence intervals.

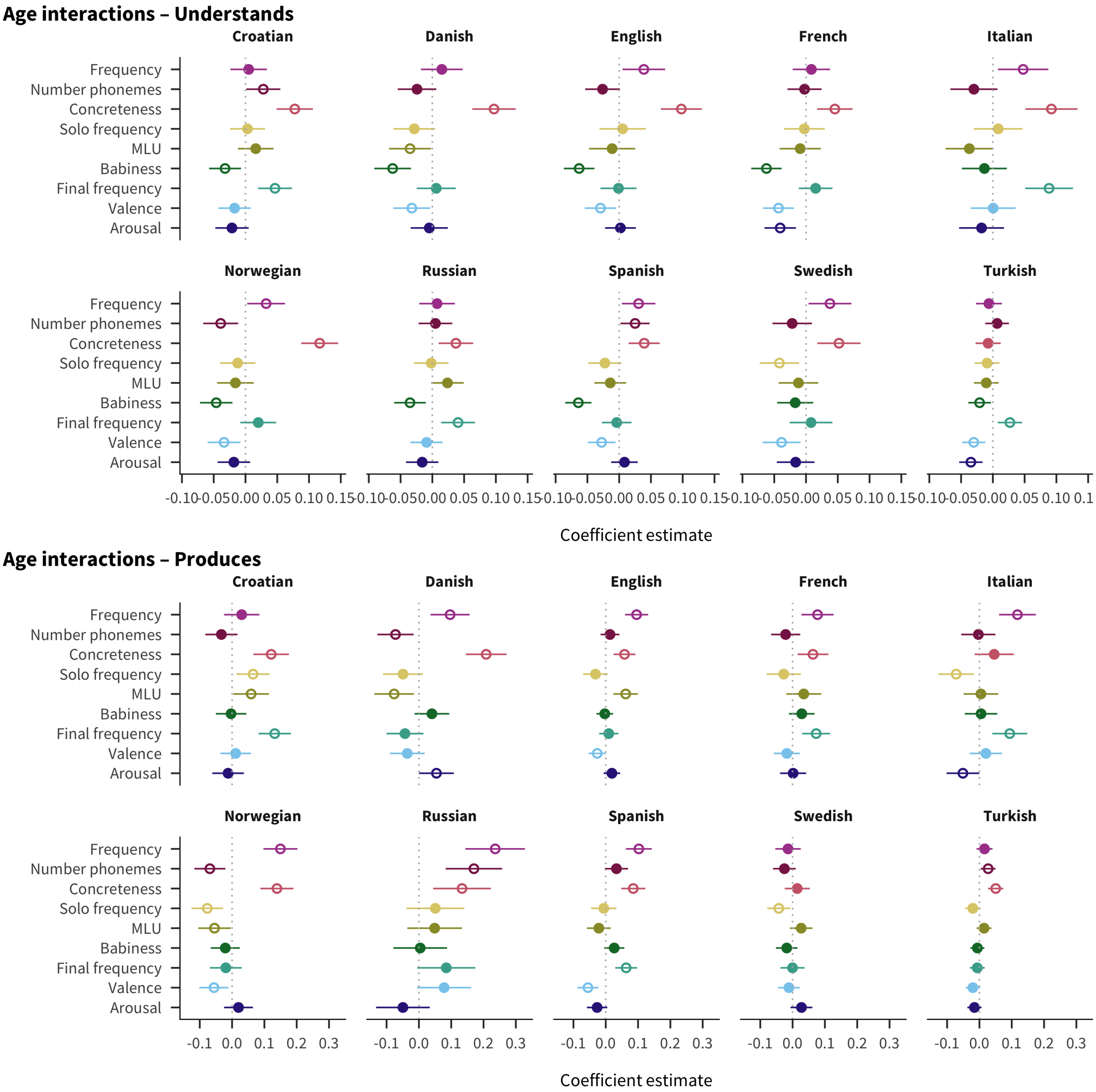

Figure SI.8. Age interactions for each language and measure with 95% confidence intervals.

Coefficients by measure

Also as a supplement to Figure 3, we show the coefficient estimates for each language, this time paired by comprehension and production.

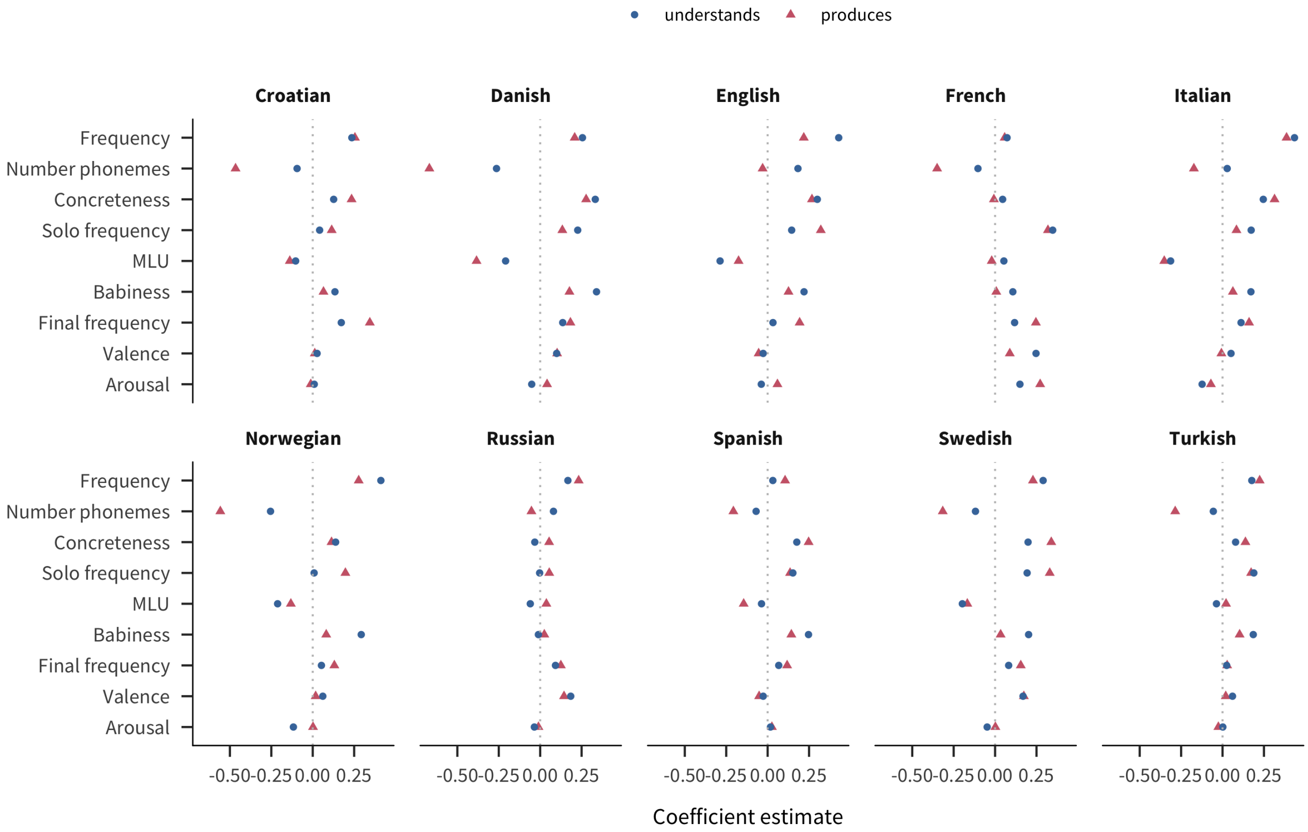

Figure SI.9. Paired coefficients for comprehension and production.

Coefficients by lexical category

As a supplement to Figure 6, we show the coefficient estimates for each language and lexical category.

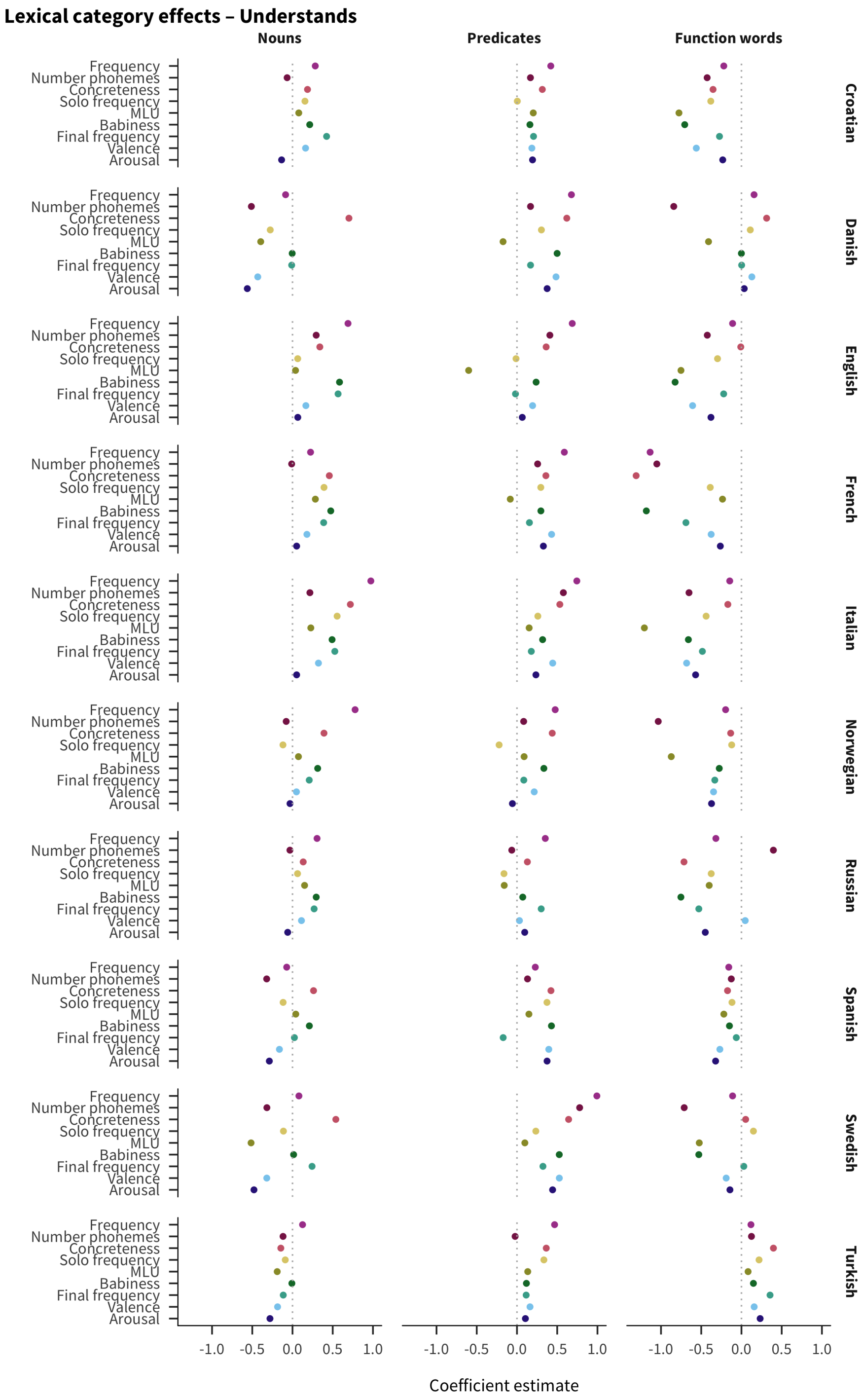

Figure SI.10. Combined effects for each lexical category (main effect of predictor + main effect of lexical category + interaction between predictor and lexical category) by language and measure.

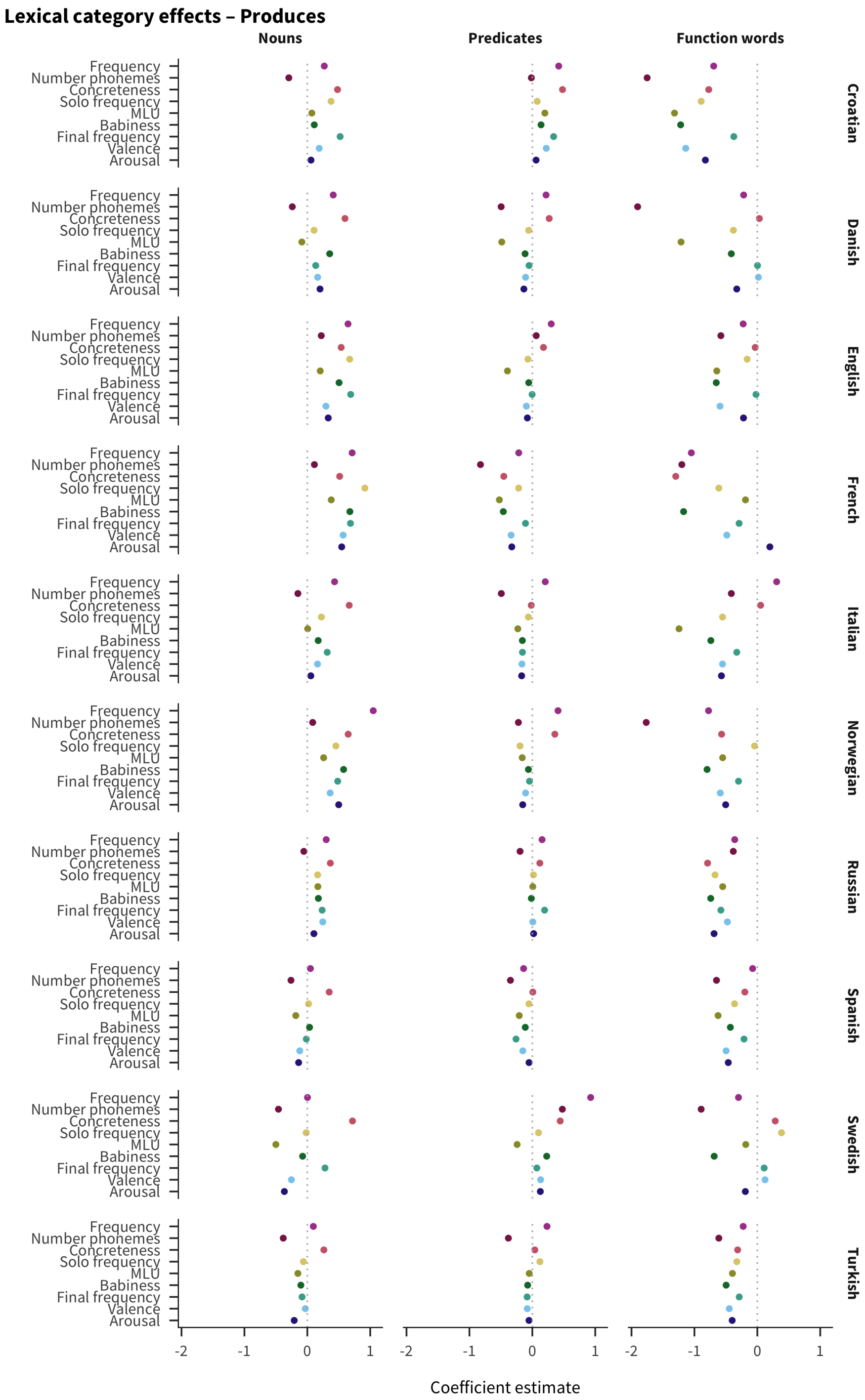

Figure SI.11. Combined effects for each lexical category (main effect of predictor + main effect of lexical category + interaction between predictor and lexical category) by language and measure.

Consistency by lexical category

As a supplement to Figure 4, we show the correlations of coefficient estimates between languages separately for each lexical category.

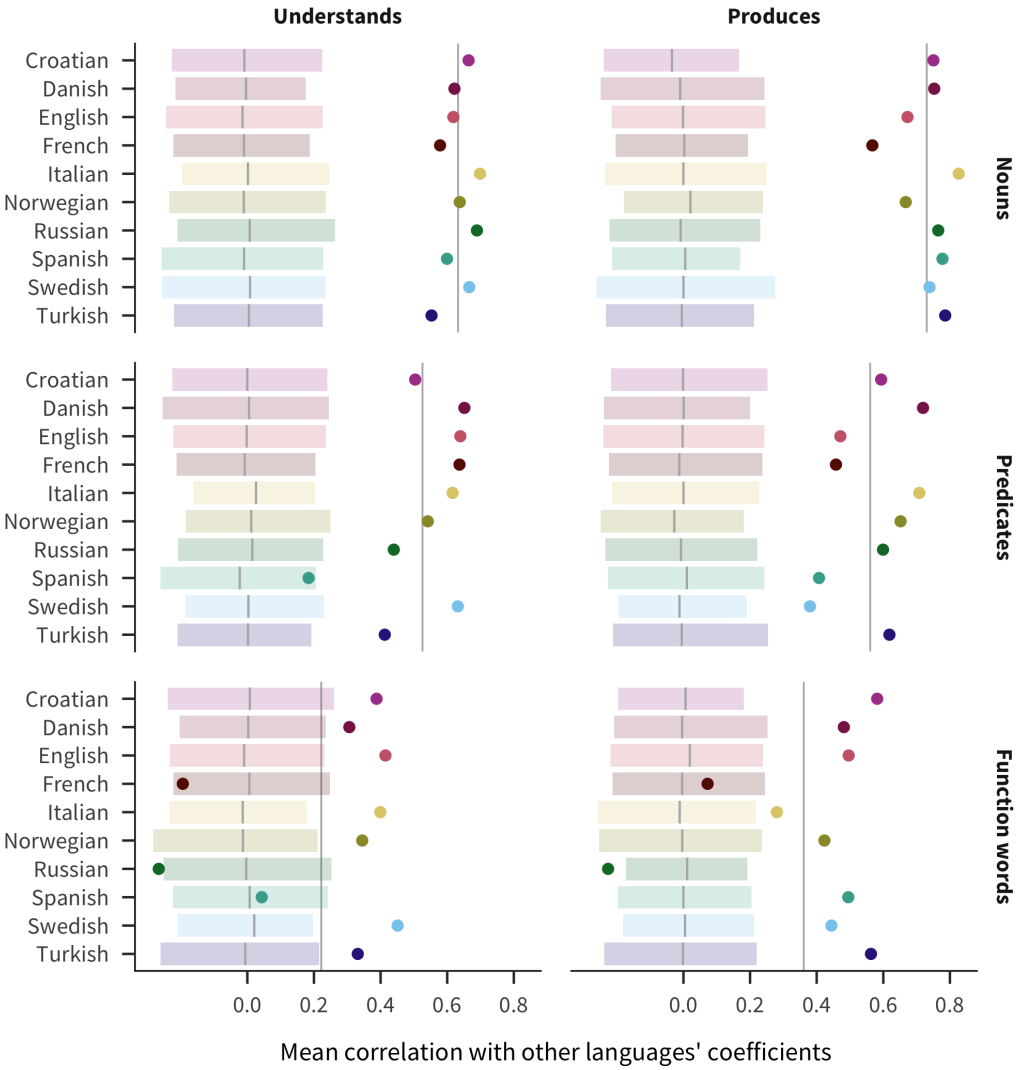

Figure SI.12. Correlations of coefficient estimates between languages within each lexical category. Each point represents the mean of one language's coefficients' correlation with each other language's coefficients, with the vertical line indicating the overall mean across languages. The shaded region and line show a bootstrapped 95% confidence interval of a randomized baseline where predictor coefficients are shuffled within language.
